# Expression of vascular endothelial growth factor (VEGF) in equine sarcoid

**DOI:** 10.1186/s12917-018-1576-z

**Published:** 2018-09-03

**Authors:** Manuela Martano, Karen Power, Brunella Restucci, Ilaria Pagano, Gennaro Altamura, Giuseppe Borzacchiello, Paola Maiolino

**Affiliations:** Department of Veterinary Medicine and Animal Productions, Naples University “Federico II”, Via F. Delpino 1, 80137 Naples, Italy

**Keywords:** Angiogenesis, BPV, Equine sarcoid, VEGF

## Abstract

**Background:**

Sarcoids are the mostcommon skin tumors in horses, characterized by rare regression, invasiveness and high recurrence following surgical intervention and Delta Papillomaviruses are widely recognized as the causative agents of the disease. In order to gain new insights into equine sarcoid development, we have evaluated, in 25 equine sarcoids, by immunohistochemistry and western blotting analysis, the expression levels of VEGF, Ki67 and bcl-2. Moreover, we have measured microvessel density and specific vessel parameters.

**Results:**

All sarcoid samples showed a strong and finely granular cytoplasmatic staining for VEGF in the majority (90%) of keratinocytes, sarcoid fibroblasts and endothelial cells. Numerous small blood vessels, immunostained with Von Willebrand factor, often appeared irregular in shape and without a distinct lumen, with mean values of microvessel area and perimeter lower than normal. Moreover, in all sarcoid samples, Ki67 immunoreactivity was moderately positive in 5–10% of dermal sarcoid fibroblasts, while Bcl2 immunoreactivity was detected in 52% of the sarcoid samples, with a weak staining in 20–50% of dermal sarcoid fibroblasts. Biochemical analysis was consistent with immunohistochemical results.

**Conclusions:**

This study has provided evidence that in equine sarcoid: VEGF was strongly expressed; the increased number of vessels was not associated with their complete maturation, probably leading to a hypoxic condition, which could increase VEGF synthesis; the levels of sarcoid fibroblasts proliferation were very low. Concluding, VEGF may have a role in equine sarcoid development, not only through the increase of angiogenesis, but also through the control of sarcoid fibroblast activity.

## Background

Sarcoids are the most common skin tumors in horses [1–3], with prevalence rates ranging from 12.9 to 67% of all equine tumors [4, 5]. They appear as benign fibroblastic skin tumors, and are characterized by rare regression, invasiveness and high recurrence following surgery [6–8]. Delta Papillomaviruses (Bovine Papillomavirus 1, Bovine Papillomavirus 2 and Bovine Papillomavirus 13) [9–11] are involved in the pathogenesis of this tumor, mainly through the biological activity of the E5 oncogene. It has also been reported that BPV alters DNA methylation status and oxidative stress parameters [12, 13].

Moreover, equine sarcoid may be considered one of the main long-term complications in the wound healing process of the horse [5, 14], consequent to abnormal fibroblast proliferation and changes in dynamics of the *extracellular matrix* (ECM) and its main components [15]. Altered turnover of the ECM deposition and degradation, as result of an altered expression of matrix metalloproteinases (MMPs) and tissue inhibitor of metalloproteinases (TIMPs), were reported as basic mechanism for these changes [15]. ECM remodeling is strictly correlated to angiogenesis [16], as MMPs production is stimulated by many of the same factors (VEGF; tumor necrosis factor alpha; *basic fibroblast growth factor*) implicated in endothelial migration and formation of new capillaries (angiogenesis) [17]. VEGF, also known as vascular permeability factor (VPF), is a member of the *platelet-derived growth factor* (*PDGF*) family of growth factors, having a potent angiogenic activity and an important role in the modulation of ECM homeostasis and remodeling [18, 19]. It is involved in numerous physiological and pathological processes, such as embryonic development [20], bone formation [21] wound healing [22] and cancer [23–27], in which it is up-regulated by oncogene expression, growth factors and hypoxia [28–30]. Moreover, it has been reported that Human Papillomavirus type 16 E6 and E7 oncoproteins may contribute to tumor angiogenesis by direct activation of the VEGF gene promoter in human lung and cervical carcinoma [31–33].

Although the general role of VEGF in angiogenic processes has been intensively studied [23–27], its specific function in sarcoid development has not been investigated, so far. In this regard, the aim of this study was to evaluate in a subset of equine sarcoids, the levels of VEGF expression in association with angiogenesis (evaluation of microvessel density and specific vessel parameters), and with Ki67 and bcl-2 expression.

## Results

### Histological features

Haematoxylin and eosin staining of examined sarcoids (*n* = 25) showed the typical histological changes in their epidermal (when present) and dermal component, as reported by Martano et al. (2016) [15], such as: hyperkeratosis and epidermal hyperplasia often accompanied by rete pegs; dermal fibroblasts usually oriented perpendicular to the basal epidermal layer in a ‘picket fence’ pattern; abundant ECM; numerous small vessels irregular in shape.

### Immunohistochemical and biochemical results

The expression patterns of VEGF, Ki67 and bcl-2 in 25 equine sarcoids and 5 normal skin samples are summarized in Table 1.

**Table 1:** Immunoreactivity scoring of VEGF, Ki67 and Bcl2 in 25 equine sarcoid samples and 5 equine normal skins

Samples	Location	VEGF	Ki67	bcl-2
		Staining Intensity	Staining Intensity	Staining Intensity
S1	neck	++	+	+
S2	limb	++	+	+/−
S3	Pectoral region	++	+	+
S4	limb	++	+	+/−
S5	head	++	+	+/−
S6	abdomen	++	+	+/−
S7	head	++	++	–
S8	Pectoral region	++	+	+/−
S9	limb	++	+	+/−
S10	(para)-Genital	++	+	+/−
S11	Limb	++	+	+
S12	abdomen	++	+	n.a.
S13	Pectoral region	++	++	+/−
S14	head	++	+	+
S15	limb	++	++	–
S16	(para)-Genital	++	+	+
S17	Limb	++	+	+/−
S18	abdomen	++	+	+
S19	Pectoral region	++	+	–
S20	neck	++	+	+
S21	limb	++	+	+
S22	(para)-Genital	++	++	n.a.
S23	Limb	++	+	+/−
S24	abdomen	++	++	–
S25	Pectoral region	++	+/−	+/−
N1	head	+/−	+	+/−
N2	abdomen	+/−	+/−	–
N3	neck	+/−	+	–
N4	limb	+/−	+/−	+/−
N5	Pectoral region	+/−	+	+/−

*N* Normal skin sample, *S* sarcoid sample, *n.a.* not assessable; − negative staining; +/− weak immunolabelling; + moderate immunolabelling; ++ extensive and strong immunolabelling

**Fig. 1:**
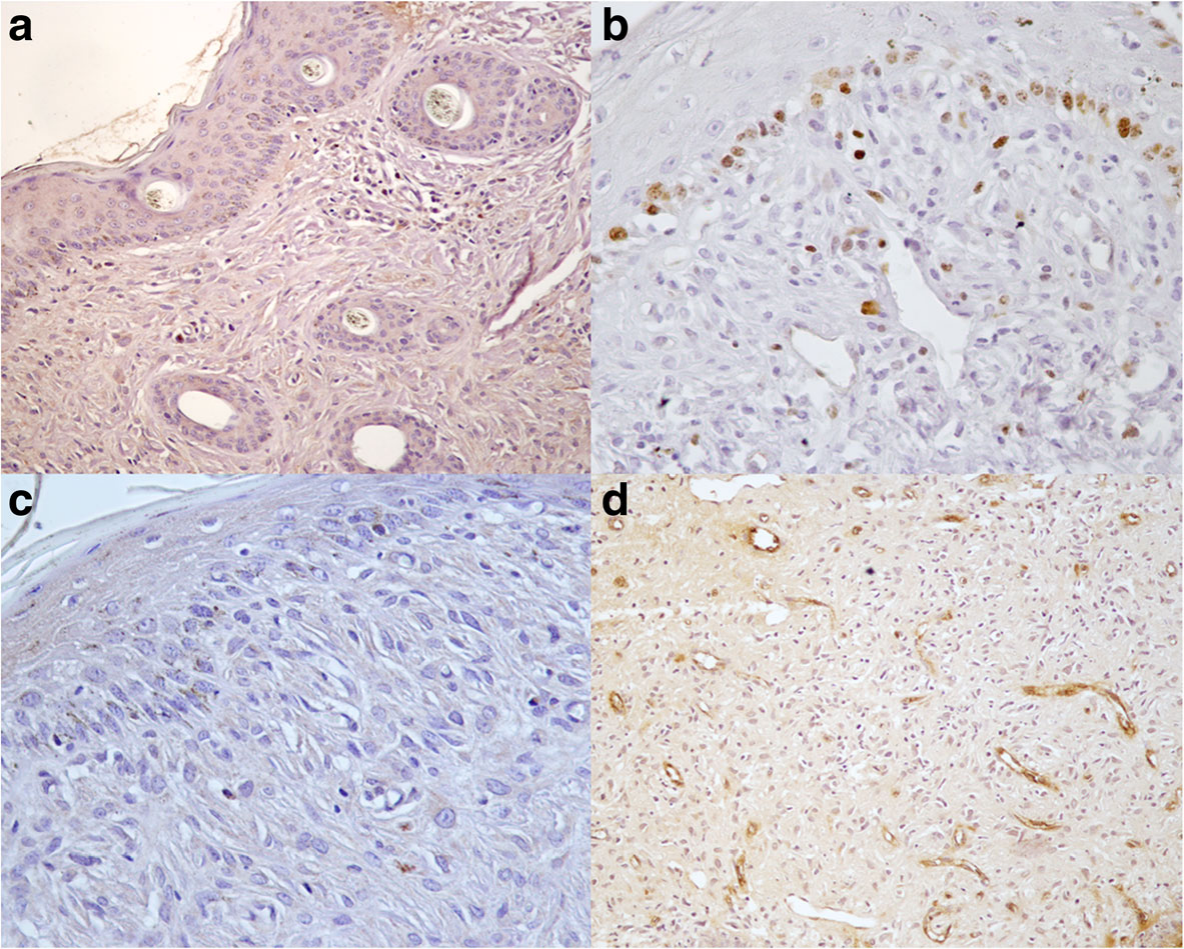
Equine normal skin. Streptavidin-biotin-peroxidase stain. **a** weak granular cytoplasmatic VEGF immunostaining was detected in the basal layer of epidermis and hair follicle while normal fibroblasts were negative 20×; **b** Ki67 immunostaining was moderate and restricted to the basal layer of epidermis 40×; **c** bcl-2 immunostaining was very weak and restricted to the basal layer of epidermis 40×; **d** blood vessels, immunolabeled with vWF, appeared regular in shape, with a distinct lumen 20×

In all normal skin samples, weak granular cytoplasmatic VEGF immunopositivity was detected predominantly in the basal layer of normal epidermis (Fig. 1a), while normal fibroblasts were negative. Ki67 immunostaining was moderate and restricted to the basal layer of epidermis and hair follicle (Fig. 1b), while bcl-2 immunostaing was very weak and in 2/5 samples was negative (Fig. 1c). Moreover, in normal skin samples, blood vessels, immunostained with vWF, appeared regular in shape and size, with a distinct lumen (Fig. 1d).

**Fig. 2:**
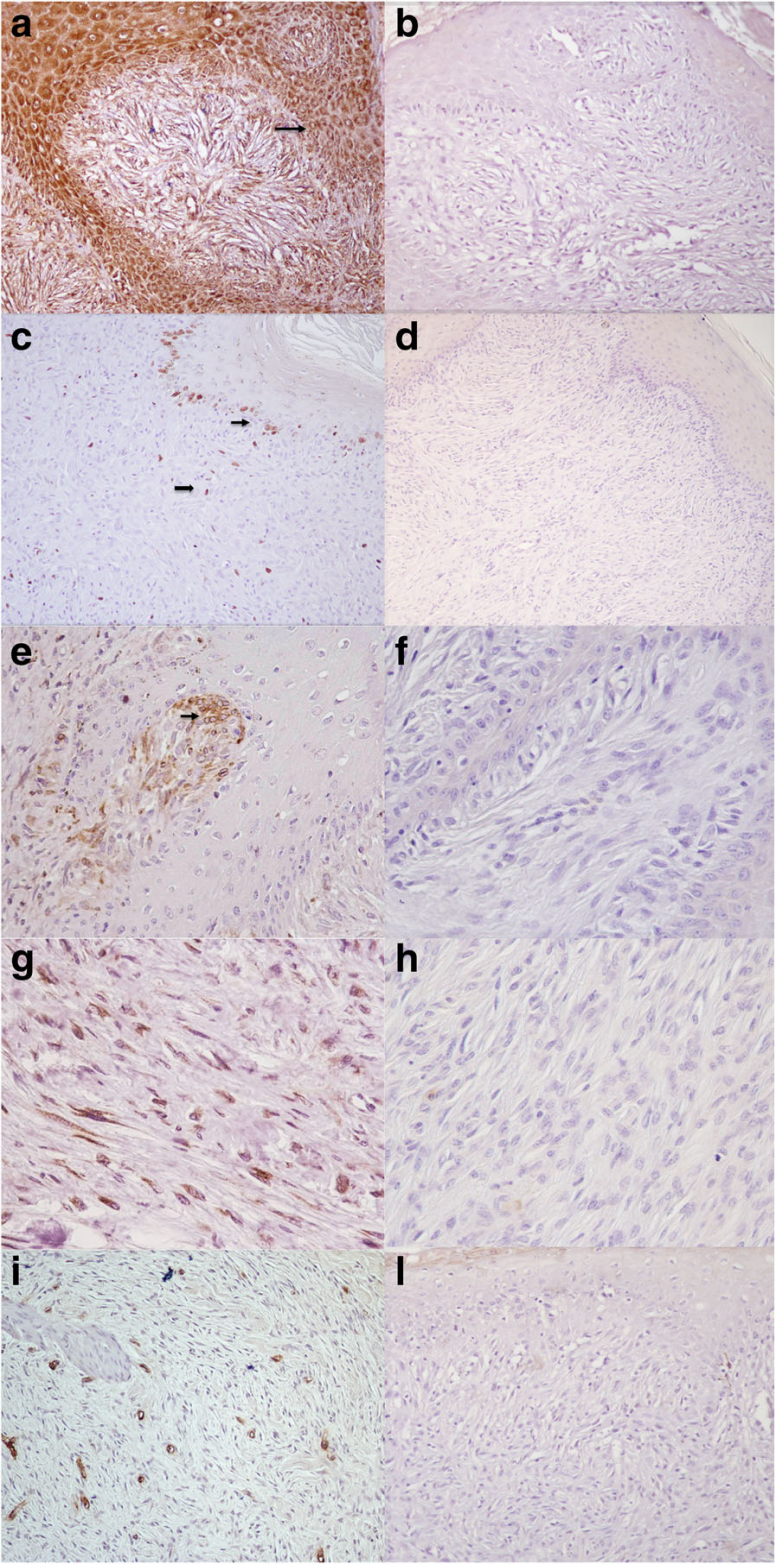
Equine sarcoid. Streptavidin-biotin-peroxidase stain. **a** Keratinocytes and fibroblasts showed a strong and finely granular cytoplasmatic staining for VEGF (arrow). 20×; **b** Secondary-only negative control for VEGF. 20×; **c** Ki67 immunoreactivity was strong in the epidermal and hair follicle basal cells (little arrow), while only few dermal fibroblasts (big arrow) were moderately positive. 20×; **d** Secondary-only negative control for Ki67. 20×; **e** Fibroblasts located immediately under the epidermis were moderately positive for bcl2 (arrow). 40×; **f** Secondary-only negative control for bcl-2. 40×; **g** bcl-2 immunoreactivity was weak in few dermal fibroblasts (arrow). 40×; **h** Secondary-only negative control for bcl-2 40×; **i** Numerous small blood vessels, immunolabeled with vWF, often appered irregular in shape and without a distinct lumen (arrow). 20×; **l** Secondary-only negative control for bcl-2. 20×

In all sarcoid samples the majority (90%) of keratinocytes, neoplastic fibroblasts and endothelial cells showed a strong and finely granular cytoplasmatic staining for VEGF (Fig. 2a; secondary-only negative control Fig. 2b) and Ki67 immunoreactivity was strong in the majority (90%) of epidermal and hair follicle basal cells and endothelial cells, while only 5–10% of neoplastic dermal fibroblasts were moderately positive (Fig. 2c; secondary-only negative control Fig. 2d). In 18 out of 25 sarcoid samples (72%) bcl-2 immunoreactivity was moderate in basal and parabasal layer of epidermis and in 20–50% of fibroblasts located immediately under the epidermis (Fig. 2e; secondary-only negative control Fig. 2f)), while the percentage of bcl-2 positive dermal fibroblasts was very low (10%) (Fig. 2g; secondary-only negative control fig. h) and the intensity of staining was weak. Moreover, in all sarcoid samples, numerous small blood vessels often appeared irregular in shape (Fig. 2i; negative control Fig.l).

**Fig. 3:**
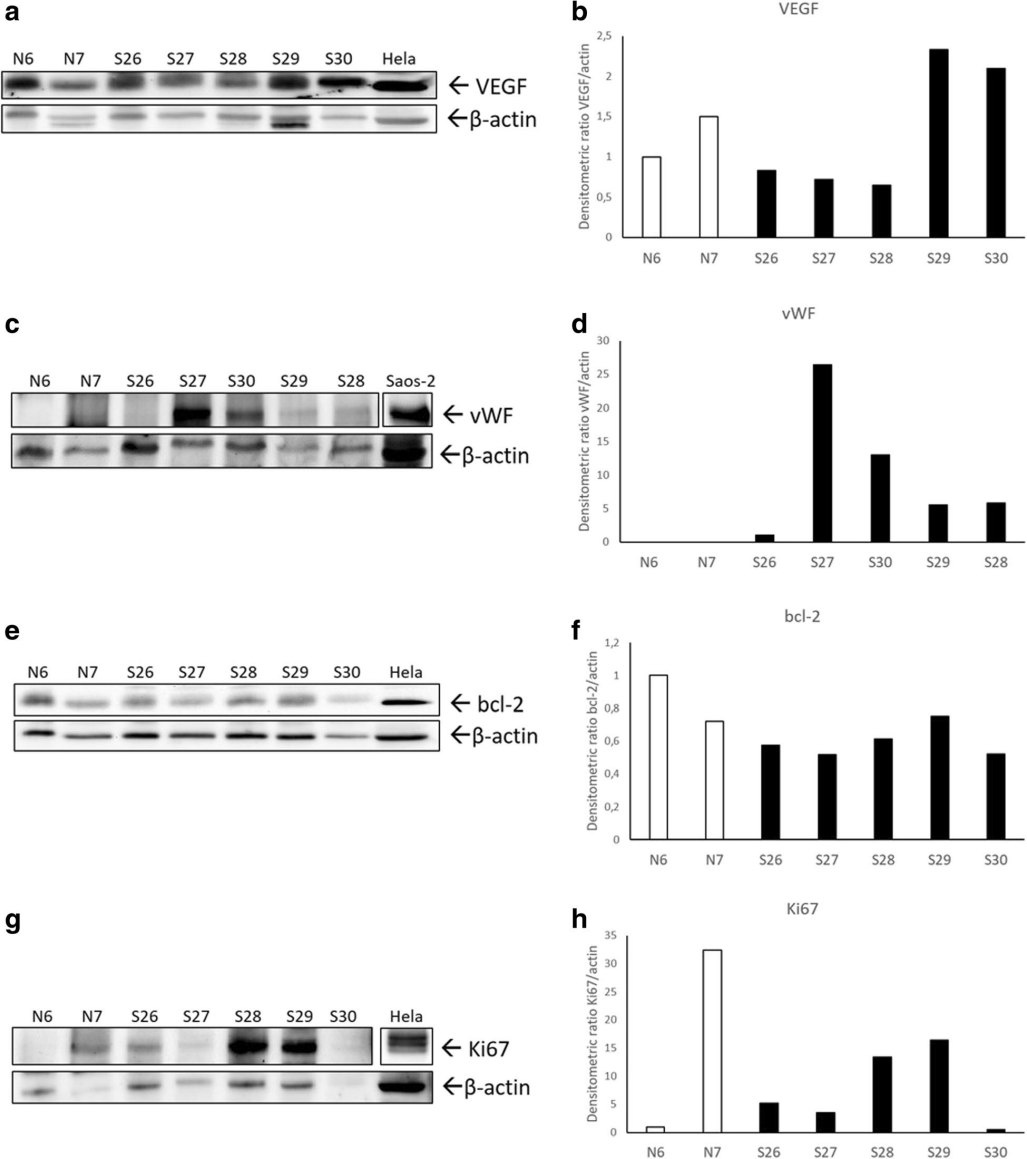
Western blotting analysis of VEGF, vWF, bcl-2 and Ki67 protein expression in equine sarcoids (S) and normal skin samples (N). **a** VEGF was expressed in all the analysed samples, with higher protein levels in S29 and S30. Hela whole cell lysate was run along with equine samples as positive control. The membrane was re-probed for β-actin to allow normalization. **b** Densitometric values were measured and expressed as VEGF/actin ratio. **c** vWF was detectable in sarcoids but not in normal skin samples. Saos-2 whole cell lysate was run along with equine samples as positive control. The Saos-2 box is cut from the same membrane at a different exposure time and properly aligned according to the molecular standard loaded onto the gel. The membrane was re-probed for β-actin to allow normalization. **d** Densitometric values were measured and expressed as vWF/actin ratio. **e** bcl-2 was expressed at variable levels in the analysed samples. Hela whole cell lysate was run along with equine samples as positive control. The membrane was re-probed for β-actin to allow normalization. **f** Densitometric values were measured and expressed as bcl-2/actin ratio. **g** Variable expression of Ki67 protein in the analysed samples. Hela whole cell lysate was run along with equine samples as positive control. The Hela box is cut from the same membrane at a different exposure time and properly aligned according to the molecular standard loaded onto the gel. The membrane was re-probed for β-actin to allow normalization. **h** Densitometric values were measured and expressed as Ki67/actin ratio

Microvessel density was lower in normal skin samples (39.5 ± 2.1) than in sarcoid samples (84.8 ± 65.06), with not statistically significant *p* value (*p* > 0.05), while vascular parameters mean values (area and perimeter) were higher in normal skins (418.02 ± 84.14; 78.01 ± 7.07) than in sarcoid samples (223.08 ± 55.92; 62.03 ± 6.08), with statistically significant *p* value (*p* <  0,05) (Table 2).

**Table 2:** Microvessel density and vascular parameters (areas and perimeters), expressed as mean (X) and standard deviation (SD), in equine sarcoids and normal skins

	Normal skin (X ± SD)	Equine sarcoids (X ± SD)	*p* values (< 0,05)
Microvessel density	39.5 ± 2.1	84.8 ± 65.06	0,3
Areas (mm^2^)	418.02 ± 84.14	223.08 ± 55.92	0,001
Perimeters (mm)	78.01 ± 7.07	62.03 ± 6.08	0,007

Western blotting analysis was performed to check the specificity of the antibodies used throughout the study (Fig. 3a-h). A band of the expected molecular size for VEGF (25 Kda), vWF (250 Kda), bcl-2 (26 Kda) and Ki67 (110 Kda) was identified in the tested samples as well as in Hela or Saos-2 cell lines used as positive control (Fig. 3a, c, e, f). The densitometric analysis of the bands normalized for β-actin revealed variable levels of VEGF protein among the samples, with higher expression in two sarcoid samples (Fig. 3b). Moreover, Von Willebrand factor protein was over-expressed in each sarcoid sample compared to each of normal skin lysates, in which the specific band was almost undetectable (Fig. 3d). The biochemical expression of bcl-2 and Ki67 was variable among the samples and no different trends between normal skin and tumor samples could be pointed out as confirmed by densitometric analysis and normalization for β-actin levels (Fig. 3f, h).

## Discussion

VEGF is a potent angiogenic factor, produced by a variety of cell types, including keratinocytes, endothelial cells, macrophages, mast cells, fibroblasts [29, 34, 35] and is involved in several types of tumors [23–27], where it has been shown to influence both tumor neovascularization and dissemination [36, 37]. Vascular endothelial growth factor can occur in at least six different isoforms, containing 121, 145, 165, 183, 189 and 206 amino acids. VEGF 121 is a freely diffusible isoform [38] and it has been proved to have a stronger angiogenic and tumorigenic activity when compared to the bigger isoforms [39–41].

In the present study, for the first time, we have demonstrated, by immunohistochemistry, the overexpression of VEGF (isoform 121) in equine sarcoid compared to normal skin, which was in part confirmed by biochemical analysis. It appeared as a strong and finely granular cytoplasmic staining pattern in the majority (> 90%) of keratinocytes, endothelial cells and sarcoid fibroblasts, suggesting a possible role in equine sarcoid development. Interestingly, in sarcoid samples we have also observed the presence of numerous small vessels, immunostained with vWF, which appeared often irregular in shape These findings were in agreement with other studies, which reported that tumor vasculature, formed under the influence of VEGF, is often structurally and functionally abnormal [42], probably as the results of the insufficient production or activation of other angiogenic factors, necessary for the formation of mature and functioning new vessels [25, 26]. These data seem to strongly support the hypothesis that in sarcoid, a suboptimal blood flow could lead to a deficient oxygen gradient within the tissue that exacerbates angiogenesis [43]. It is well known that tissue mild hypoxia, an almost universal condition within wound healing, from which sarcoid could origin, is an effective inducer of VEGF synthesis [44]. Therefore, in sarcoid, a vicious circle may occur in which hypoxia upregulates VEGF synthesis, leading to an insufficient vascularization, which in turn probably exacerbates hypoxia. Despite numerous studies characterizing its angiogenic property, data on the role of VEGF in ECM homeostasis and remodeling are still scanty.

For this reason, we speculate that, in equine sarcoid, VEGF could be implicated not only in angiogenesis but also in ECM homeostasis and remodeling, through a deregulation of fibroblasts proliferation and apoptosis in a possible hypoxic condition. This mechanism was also reported during endochondral bone formation, in which VEGF couples hypertrophic cartilage remodeling, ossification and angiogenesis [21]. In this regard, a very low fibroblast growth rate has been reported in equine sarcoid, along with a low frequency of p53 mutations [45]. These last were generally associated with abnormalities in apoptotic pathways rather than abnormal cell cycle control mechanism [46].

In our sarcoids samples, the percentage of Ki67 positive fibroblasts ranged from 5 to 10% and, in agreement with previous studies [46–48], these results confirm that the rate of fibroblast proliferation in equine sarcoid was very low. Moreover, these observations correspond to the clinical evidence that equine sarcoids are slow growing tumors and can remain quiescent for years [47]. IHC and biochemical data did not prove an overall differential expression of Ki67 and bcl-2 in sarcoids compared to normal skin; however, the more frequent localization of BPV-positive cells, right near the dermo-epidermal junction [49, 50], could explain the higher expression of Ki67 and bcl-2 observed in epithelial portion and in dermo-epidermal junction.

## Conclusion

Concluding, in this study we hypothesized that VEGF might have a role in sarcoid development, by altering ECM homeostasis, through a selection of a quiescent population of fibroblast, leading to an impaired degradation and excessive accumulation of ECM [14]. This also seems to be also supported by our previous study, which documented that the excessive and progressive deposition of connective tissue (collagen) in sarcoid is not only the result of elevated synthesis by fibroblasts, but it is also caused by a deficiency in matrix degradation due to an alterated expression of MMPs and TIMPs [15].

Currently, despite the numerous *sarcoid treatment* choices available, *not* all *sarcoids* are responsive. Hence, the importance of a better knowledge of equine sarcoid development. Further studies in the near future are needed to investigate the association between equine sarcoid and tissue hypoxia, which could have a crucial role for the development of new therapeutic strategies.

## Methods

### Tumor samples

A total of 25 (S1-S25) equine sarcoids (each from a different horse), clinically identified based on their gross morphology according to Pascoe and Knottenbelt [51], were obtained from affected horses, which underwent surgery routinely, adhering to a high standard (best practice) of veterinary care, after informed written consent of the owners, and according to Directives 2010/63/EU (art. 1 c. 4) and 2010/63/EU. Tumors were localized on the neck (2), limbs (8), pectoral region (5), head (3), abdomen (4), (para)-genital (3) (Table 1). Sarcoid tissues used in this study were known to be BPV positive [48].

As controls, tissues from 5 normal skin BPV positive samples (N1-N5), were taken during necropsy from five healthy horses. We did not seek committee approval as all samples analyzed were not collected as part of experimental clinical veterinary practices but as routine diagnosis and treatment, according to Directive 2010/63/EU (art. 1 c. 4) on protection of animals used for scientific purposes. All samples were 10% formalin fixed, paraffin-embedded for routine histological processing and stained with haematoxylin and eosin for light microscopy study.

Additional five sarcoids (S26-S30) and 2 normal skin samples (N6-N7) were collected as above and immediately frozen at − 80 °C in order to allow Western blotting analysis.

### Immunohistochemistry

Paraffin sections of equine sarcoids (S1-S25) and normal skin (N1-N5) from healthy horses were dewaxed in xylene, dehydrated in graded alcohols and washed in 0.01 M phosphate-buffered saline (PBS), pH 7.2–7.4. Endogenous peroxidase was blocked with hydrogen peroxide 0.3% in absolute methanol for 30 min. The immunohistochemical procedure (streptavidin biotin-peroxidase method) for the detection of VEGF, bcl-2, Ki67 and vWF was the same as that used by the authors in a previous study [15]. The immunolabelling procedure included negative control sections incubated with PBS instead of the primary antibodies (Fig. 2b, d, f, h, l). Primary antibodies, dilutions and antigen retrieval techniques used in this study are listed in Table 3. Primary antibodies were diluted in phosphate-buffered saline (PBS) and applied overnight at 4 °C. After 2 washes in PBS, MACH 1 mouse probe (Biocare Medical, LLC, Concord, CA, USA) was applied for 20 min at room temperature. After, MACH-1 Universal HPR-Polymer (Biocare Medical) was applied for 30 min at room temperature. To reveal immunolabelling, diaminobenzidinetetrahydrochloride was used as a chromogen, and haematoxylin as counterstain.

**Table 3:** List of primary antibodies used for immunohistochemistry and western blotting analysis

Antibody	Manufacturer	Clone	Host species	Antigen retriever	IHC Dilution	WB Dilution
vWF	DAKO	Polyclonal	Rabbit	0.05% pepsin in 0.01 M HCl (pH 2) at 37 ° C for 15 min	1:400	1:1000
bcl-2	DAKO	124	mouse	Heated Citrate buffer, ph 6.0, 30 min	1:100	1:1000
VEGF	Thermoscientific	JH121	mouse	Heated Citrate buffer, ph 6.0, 30 min,	1:200	1:200
Ki67	Santa Cruz	D-6	mouse	Heated Citrate buffer, ph 6.0, 30 min,	1:100	1: 1000

### Scoring of immunoreactivity

The intensity of immunolabelling in each specimen, for each antibody, was scored by two independent observers (MM and KP) under blinded conditions, as performed in a previous study [52]. Briefly, for each sample the immunoreactivity was scored from negative to strong, as follows: n.a., not assessable; − negative staining; +/− weak immunolabelling; + moderate immunolabelling; ++ extensive and strong immunolabelling. Moreover, the number of positively-labelled cells was established by counting 1.000 cells in 10 fields at 400× magnification (40× objective 10× ocular) and results were expressed as percentage.

### Microvessel density and vascular parameter measurements

Microvessel density (number of vessels per mm^2^) and vascular parameter measurements (vessel area and perimeter) were performed on vWF immunostained sections, using a free image analysis software (Image J). For each tumor 10 fields, randomly selected, were captured at 400× magnification (40× objective 10× ocular) with a microscope (E-400; Nikon Eclipse, Tokyo, Japan) coupled to a videocamera (TKC1380E; JVC, Tokyo, Japan), stored in the digital memory, and shown on the monitor. The fields were examined and manual outlining of immunolabelled microvessels was performed; microvessel density, areas, perimeters were then calculated based on image analysis, and results were expressed as mean (X) and standard deviation values (±SD) (Table 2). Statistical assessment was performed using a Student t-test by analysis of variance (ANOVA) and *p*-value< 0.05 with 95% confidence interval (CI) was considered statistically significant.

### Protein extraction and SDS PAGE/western blotting

Tissue samples N6-N7 and T26-T30 were homogenized in ice-cold RIPA buffer (50 mM Tris-HCl, pH 7.5, 150 mM NaCl, 1% Triton X100, 1 mM EDTA, deoxycholate 0.25%) added with phosphatase and protease inhibitor cocktails (Sigma-Aldrich, Milan, Italy) by using TissueLyser machinery (Qiagen, Milan, Italy) according to manufacturer’s protocol. Tissue homogenates were incubated on a rocking wheel for 30′ at 4 °C. Protein extracts were clarified by high-speed centrifugation at 4 °C for 30’and total proteins were quantified by Bradford assay (Bio-Rad, Bio-Rad Laboratories, Segrate (MI), Italy). Equal amounts of lysates were resuspended in Laemmli sample buffer (Bio-Rad), boiled and analyzed by denaturing polyacrylamide gel electrophoresis (SDS PAGE). Proteins were blotted from gel onto nitrocellulose membranes using TransBlot Turbo apparatus (Bio-Rad, Bio-Rad Laboratories, Segrate (MI), Italy). The membranes were blocked with 5% non-fat dry milk in Tris buffered saline (10 mM Tris-HCl, pH 7.4, 165 mM NaCl) with 0.1% tween (TTBS), for 1 h (h) at room temperature (RT) and incubated over night at 4 °C with the primary antibodies at the indicated dilutions: anti- Bcl2 (1:1000), anti-VEGF (1:200), anti-Ki67 (1:1000), anti-vWF (1:1000). After four washing steps of 10 minutes, appropriate peroxidase-conjugated secondary antibodies were applied for 1 h at RT at 1:2000 dilution. Following further four washings in TTBS, bound antibodies were visualized by enhanced chemiluminescence (Cyanagen, Bologna, Italy). The blots were stripped and reprobed against mouse anti-β-actin antibody (CP01, Calbiochem, San Diego, CA, USA) (1:500) to allow normalization. Protein levels were quantitatively estimated by densitometric analysis using ChemiDoc gel scanner (Bio-Rad) equipped with a densitometric workstation (Image Lab software, (Bio-Rad Laboratories, Segrate (MI), Italy). Proteins concentrations were normalized to the β-actin levels.

## Abbreviations

bcl-2: B-cell lymphoma 2
BPV: Bovine papillomavirus
BPV-2: Bovine papillomavirus type-2
ECM: Extracellular Matrix
MMP: matrix metalloproteinase
N: normal skin
PBS: phosphate-buffered saline
PDGF: platelet-derived growth factor
S: samples T: sarcoid samples
TIMP: Tissue inhibitor of metalloproteinase
VEGF: Vascular endothelial growth factor
vWF: Von Willebrandt factor
